# Supplementary material for: Computer modelling reveals new conformers of the ATP binding loop of Na+/K+-ATPase involved in the transphosphorylation process of the sodium pump
Source: PeerJ. 2017 Mar 14;5:e3087. doi: 10.7717/peerj.3087 (PMC5354106; doi:10.7717/peerj.3087)
Supplement: Figure S1 [file peerj-05-3087-s001.pdf]

```
3B8EA   TLTAKRMARKNCLVKNLEAVETLGSTSTICSDKTGTLTQNRMTVAHMWSDNQIHEADTTENQSGVSFDKTSATWLALSRIAGLCNRAVFQANQENLPILK   100
3B8EC   TLTAKRMARKNCLVKNLEAVETLGSTSTICSDKTGTLTQNRMTVAHMWSDNQIHEADTTENQSGVSFDKTSATWLALSRIAGLCNRAVFQANQENLPILK   100
3KDPA   TLTAKRMARKNCLVKNLEAVETLGSTSTICSDKTGTLTQNRMTVAHMWSDNQIHEADTTENQSGVSFDKTSATWLALSRIAGLCNRAVFQANQENLPILK   100
3KDPC   TLTAKRMARKNCLVKNLEAVETLGSTSTICSDKTGTLTQNRMTVAHMWSDNQIHEADTTENQSGVSFDKTSATWLALSRIAGLCNRAVFQANQENLPILK   100
P50993  TLTAKRMARKNCLVKNLEAVETLGSTSTICSDKTGTLTQNRMTVAHMWFDNQIHEADTTEDQSGATFDKRSPTWTALSRIAGLCNRAVFKAGQENISVSK   100

3B8EA   RAVAGDASESALLKCIELCCGSVKEMRERYTKIVEIPFNSTNKYQLSIHKNPNTAEPRHLLVMKGAPERILDRCSSILIHGKEQPLDEELKDAFQNAYLE   200
3B8EC   RAVAGDASESALLKCIELCCGSVKEMRERYTKIVEIPFNSTNKYQLSIHKNPNTAEPRHLLVMKGAPERILDRCSSILIHGKEQPLDEELKDAFQNAYLE   200
3KDPA   RAVAGDASESALLKCIELCCGSVKEMRERYTKIVEIPFNSTNKYQLSIHKNPNTAEPRHLLVMKGAPERILDRCSSILIHGKEQPLDEELKDAFQNAYLE   200
3KDPC   RAVAGDASESALLKCIELCCGSVKEMRERYTKIVEIPFNSTNKYQLSIHKNPNTAEPRHLLVMKGAPERILDRCSSILIHGKEQPLDEELKDAFQNAYLE   200
P50993  RDTAGDASESALLKCIELSCGSVRKMRDRNPKVAEIPFNSTNKYQLSIHEREDSPQS.HVLVMKGAPERILDRCSTILVQGKEIPLDKEMQDAFQNAYME   199

3B8EA   LGGLGERVLGFCHLFLPDEQFPEGFQFDTDDVNFPLDNLCFVGLISMIDPPRAAVPDAVGKCRSAGIKVIMVTGDHPITAKAIAKGVGIISEGNETVEDI   300
3B8EC   LGGLGERVLGFCHLFLPDEQFPEGFQFDTDDVNFPLDNLCFVGLISMIDPPRAAVPDAVGKCRSAGIKVIMVTGDHPITAKAIAKGVGIISEGNETVEDI   300
3KDPA   LGGLGERVLGFCHLFLPDEQFPEGFQFDTDDVNFPLDNLCFVGLISMIDPPRAAVPDAVGKCRSAGIKVIMVTGDHPITAKAIAKGVGIISEGNETVEDI   300
3KDPC   LGGLGERVLGFCHLFLPDEQFPEGFQFDTDDVNFPLDNLCFVGLISMIDPPRAAVPDAVGKCRSAGIKVIMVTGDHPITAKAIAKGVGIISEGNETVEDI   300
P50993  LGGLGERVLGFCQLNLPSGKFPRGFKFDTDELNFPTEKLCFVGLMSMIDPPRAAVPDAVGKCRSAGIKVIMVTGDHPITAKAIAKGVGIISEGNETVEDI   299

3B8EA   AARLNIPVSQVNPRDAKACVVHGSDLKDMTSEQLDDILKYHTEIVFARTSPQQKLIIVEGCQRQGAIVAVTGDGVNDSPASKKADIGVAMGIAGSDVSKQ   400
3B8EC   AARLNIPVSQVNPRDAKACVVHGSDLKDMTSEQLDDILKYHTEIVFARTSPQQKLIIVEGCQRQGAIVAVTGDGVNDSPASKKADIGVAMGIAGSDVSKQ   400
3KDPA   AARLNIPVSQVNPRDAKACVVHGSDLKDMTSEQLDDILKYHTEIVFARTSPQQKLIIVEGCQRQGAIVAVTGDGVNDSPASKKADIGVAMGIAGSDVSKQ   400
3KDPC   AARLNIPVSQVNPRDAKACVVHGSDLKDMTSEQLDDILKYHTEIVFARTSPQQKLIIVEGCQRQGAIVAVTGDGVNDSPASKKADIGVAMGIAGSDVSKQ   400
P50993  AARLNIPMSQVNPREAKACVVHGSDLKDMTSEQLDEILKNHTEIVFARTSPQQKLIIVEGCQRQGAIVAVTGDGVNDSPALKKADIGIAMGISGSDVSKQ   399

3B8EA   AADMILLDDNFASIVTGVEEGRLI   424
3B8EC   AADMILLDDNFASIVTGVEEGRLI   424
3KDPA   AADMILLDDNFASIVTGVEEGRLI   424
3KDPC   AADMILLDDNFASIVTGVEEGRLI   424
P50993  AADMILLDDNFASIVTGVEEGRLI   423
```
