# Supplementary material for: Computer modelling reveals new conformers of the ATP binding loop of Na+/K+-ATPase involved in the transphosphorylation process of the sodium pump
Source: PeerJ. 2017 Mar 14;5:e3087. doi: 10.7717/peerj.3087 (PMC5354106; doi:10.7717/peerj.3087)
Supplement: Figure S2 [file peerj-05-3087-s002.pdf]

```
3WGUA   TLTAKRMARKNCLVKNLEAVETLGSTSTICSDKTGTLTQNRMTVAHMWFDNQIHEADTTENQSGVSFDKTSATWLALSRIAGLCNRAVFQANQENLPILK   100
3WGUC   TLTAKRMARKNCLVKNLEAVETLGSTSTICSDKTGTLTQNRMTVAHMWFDNQIHEADTTENQSGVSFDKTSATWLALSRIAGLCNRAVFQANQENLPILK   100
3WGVA   TLTAKRMARKNCLVKNLEAVETLGSTSTICSDKTGTLTQNRMTVAHMWFDNQIHEADTTENQSGVSFDKTSATWLALSRIAGLCNRAVFQANQENLPILK   100
3WGVC   TLTAKRMARKNCLVKNLEAVETLGSTSTICSDKTGTLTQNRMTVAHMWFDNQIHEADTTENQSGVSFDKTSATWLALSRIAGLCNRAVFQANQENLPILK   100
4HQJA   TLTAKRMARKNCLVKNLEAVETLGSTSTICSDKTGTLTQNRMTVAHMWSDNQIHEADTTENQSGVSFDKTSATWLALSRIAGLCNRAVFQANQENLPILK   100
4HQJC   TLTAKRMARKNCLVKNLEAVETLGSTSTICSDKTGTLTQNRMTVAHMWSDNQIHEADTTENQSGVSFDKTSATWLALSRIAGLCNRAVFQANQENLPILK   100
P50993  TLTAKRMARKNCLVKNLEAVETLGSTSTICSDKTGTLTQNRMTVAHMWFDNQIHEADTTEDQSGATFDKRSPTWTALSRIAGLCNRAVFKAGQENISVSK   100

3WGUA   RAVAGDASESALLKCIELCCGSVKEMRERYTKIVEIPFNSTNKYQLSIHKNPNTAEPRHLLVMKGAPERILDRCSSILIHGKEQPLDEELKDAFQNAYLE   200
3WGUC   RAVAGDASESALLKCIELCCGSVKEMRERYTKIVEIPFNSTNKYQLSIHKNPNTAEPRHLLVMKGAPERILDRCSSILIHGKEQPLDEELKDAFQNAYLE   200
3WGVA   RAVAGDASESALLKCIELCCGSVKEMRERYTKIVEIPFNSTNKYQLSIHKNPNTAEPRHLLVMKGAPERILDRCSSILIHGKEQPLDEELKDAFQNAYLE   200
3WGVC   RAVAGDASESALLKCIELCCGSVKEMRERYTKIVEIPFNSTNKYQLSIHKNPNTAEPRHLLVMKGAPERILDRCSSILIHGKEQPLDEELKDAFQNAYLE   200
4HQJA   RAVAGDASESALLKCIELCCGSVKEMRERYTKIVEIPFNSTNKYQLSIHKNPNTAEPRHLLVMKGAPERILDRCSSILIHGKEQPLDEELKDAFQNAYLE   200
4HQJC   RAVAGDASESALLKCIELCCGSVKEMRERYTKIVEIPFNSTNKYQLSIHKNPNTAEPRHLLVMKGAPERILDRCSSILIHGKEQPLDEELKDAFQNAYLE   200
P50993  RDTAGDASESALLKCIELSCGSVRKMRDRNPKVAEIPFNSTNKYQLSIHEREDSPQS.HVLVMKGAPERILDRCSTILVQGKEIPLDKEMQDAFQNAYME   199

3WGUA   LGGLGERVLGFCHLFLPDEQFPEGFQFDTDDVNFPLDNLCFVGLISMIDPPRAAVPDAVGKCRSAGIKVIMVTGDHPITAKAIAKGVGIISEGNETVEDI   300
3WGUC   LGGLGERVLGFCHLFLPDEQFPEGFQFDTDDVNFPLDNLCFVGLISMIDPPRAAVPDAVGKCRSAGIKVIMVTGDHPITAKAIAKGVGIISEGNETVEDI   300
3WGVA   LGGLGERVLGFCHLFLPDEQFPEGFQFDTDDVNFPLDNLCFVGLISMIDPPRAAVPDAVGKCRSAGIKVIMVTGDHPITAKAIAKGVGIISEGNETVEDI   300
3WGVC   LGGLGERVLGFCHLFLPDEQFPEGFQFDTDDVNFPLDNLCFVGLISMIDPPRAAVPDAVGKCRSAGIKVIMVTGDHPITAKAIAKGVGIISEGNETVEDI   300
4HQJA   LGGLGERVLGFCHLFLPDEQFPEGFQFDTDDVNFPLDNLCFVGLISMIDPPRAAVPDAVGKCRSAGIKVIMVTGDHPITAKAIAKGVGIISEGNETVEDI   300
4HQJC   LGGLGERVLGFCHLFLPDEQFPEGFQFDTDDVNFPLDNLCFVGLISMIDPPRAAVPDAVGKCRSAGIKVIMVTGDHPITAKAIAKGVGIISEGNETVEDI   300
P50993  LGGLGERVLGFCQLNLPSGKFPRGFKFDTDELNFPTEKLCFVGLMSMIDPPRAAVPDAVGKCRSAGIKVIMVTGDHPITAKAIAKGVGIISEGNETVEDI   299

3WGUA   AARLNIPVSQVNPRDAKACVVHGSDLKDMTSEQLDDILKYHTEIVFARTSPQQKLIIVEGCQRQGAIVAVTGDGVNDSPALKKADIGVAMGIAGSDVSKQ   400
3WGUC   AARLNIPVSQVNPRDAKACVVHGSDLKDMTSEQLDDILKYHTEIVFARTSPQQKLIIVEGCQRQGAIVAVTGDGVNDSPALKKADIGVAMGIAGSDVSKQ   400
3WGVA   AARLNIPVSQVNPRDAKACVVHGSDLKDMTSEQLDDILKYHTEIVFARTSPQQKLIIVEGCQRQGAIVAVTGDGVNDSPALKKADIGVAMGIAGSDVSKQ   400
3WGVC   AARLNIPVSQVNPRDAKACVVHGSDLKDMTSEQLDDILKYHTEIVFARTSPQQKLIIVEGCQRQGAIVAVTGDGVNDSPALKKADIGVAMGIAGSDVSKQ   400
4HQJA   AARLNIPVSQVNPRDAKACVVHGSDLKDMTSEQLDDILKYHTEIVFARTSPQQKLIIVEGCQRQGAIVAVTGDGVNDSPASKKADIGVAMGIAGSDVSKQ   400
4HQJC   AARLNIPVSQVNPRDAKACVVHGSDLKDMTSEQLDDILKYHTEIVFARTSPQQKLIIVEGCQRQGAIVAVTGDGVNDSPASKKADIGVAMGIAGSDVSKQ   400
P50993  AARLNIPMSQVNPREAKACVVHGSDLKDMTSEQLDEILKNHTEIVFARTSPQQKLIIVEGCQRQGAIVAVTGDGVNDSPALKKADIGIAMGISGSDVSKQ   399

3WGUA   AADMILLDDNFASIVTGVEEGRLI   424
3WGUC   AADMILLDDNFASIVTGVEEGRLI   424
3WGVA   AADMILLDDNFASIVTGVEEGRLI   424
3WGVC   AADMILLDDNFASIVTGVEEGRLI   424
4HQJA   AADMILLDDNFASIVTGVEEGRLI   424
4HQJC   AADMILLDDNFASIVTGVEEGRLI   424
P50993  AADMILLDDNFASIVTGVEEGRLI   423
```
