# Supplementary material for: Computer modelling reveals new conformers of the ATP binding loop of Na+/K+-ATPase involved in the transphosphorylation process of the sodium pump
Source: PeerJ. 2017 Mar 14;5:e3087. doi: 10.7717/peerj.3087 (PMC5354106; doi:10.7717/peerj.3087)
Supplement: Supplemental Information 1 [file peerj-05-3087-s007.docx]

Supplement material

Computer modelling reveals new conformers of the ATP binding loop of Na^+^/K^+^-ATPase involved in the transphosphorylation process of the sodium pump

G. Tejral^1,2^, B. Sopko^3^, A. Necas^4^, W. Schoner^5^ and E. Amler^1,2^

^1^Laboratory of Tissue Engineering, Institute of Experimental Medicine, Academy of Sciences of the Czech Republic, Prague, Czech Republic,

^2^Department of Biophysics, 2^nd^ Faculty of Medicine, Charles University Prague, Czech Republic,

^3^Department of Medical Chemistry and Clinical Biochemistry, 2^nd^ Faculty of Medicine, Charles University and Motol University Hospital, Prague, Czech Republic,

^4^Small Animal Clinic, Faculty of Veterinary Medicine, University of Veterinary and Pharmaceutical Sciences Brno, Brno, Czech Republic

^5^Institute of Biochemistry and Endocrinology, University of Giessen, Giessen, Germany

Corresponding author: Evžen Amler, Prof., PhD., Charles University in Prague, 2^nd^ Faculty of Medicine, Department of Biophysics, V Úvalu 84, 150 06 Prague 5, Czech Republic. Phone: +420 257 296 350, Fax: +420 257 296 355, E-mail: evzen.amler@lfmotol.cuni.cz

# Methods

***Comparative modeling of the open conformation***

For modeling, known structures of Na^+^/K^+^-ATPase deposited at the RCSB Protein Data Bank (http://www.pdb.org/) were used. In order to create the model based on the above mentioned sequence, the solved crystal structures of Na^+^/K^+^-ATPase with RCSB Protein Data Bank (http://www.pdb.org/) accession codes 3B8E ([Morth, Pedersen et al. 2007](#_ENREF_4)) and 3KDP([Morth, Pedersen et al. 2007](#_ENREF_4)) were used as the templates for our modeling. The multialignment of the chosen target (P50993, AT1A2_HUMAN) sequence and the two templates (3B8E, 3KDP) for open conformation was prepared by MODELLER program (salign module) – see Figure S-1.

***Comparative modeling of the closed conformation***

As in the previous comparative modeling procedure, we have used the sequence P50993 (AT1A2_HUMAN) for modeling of Na^+^/K^+^-ATPase in the closed conformation. However, the solved crystal structures of the RCSB Protein Data Bank (http://www.pdb.org/ - accession codes 3WGU, 3WGV and 4HQJ([Kanai, Ogawa et al. 2013](#_ENREF_3), [Nyblom, Poulsen et al. 2013](#_ENREF_5))) were used as the templates for our modeling – see Figure S-2.

***Molecular dynamics***

The PME method ([Darden, York et al. 1993](#_ENREF_2)) with a length parameter of 1 nm was used to describe Coulomb type electrostatic interactions and the cut-off method with a length parameter of 1 nm for the calculation of van der Waals interactions. As the first step of the MD simulation, the system of protein and water was energetically optimized using the method of steepest descents, followed by a conjugate gradient minimization algorithm with maximum 2.5x10^4^ steps (see supplement material). The Berendsen coupling method ([Berendsen, Postma et al. 1984](#_ENREF_1)) was employed for the temperature and pressure coupling of a system to reflect the reference temperature of 300K and the pressure of 1 bar. The leap-frog integration with 10^4^ steps was used for stabilization, with integration step of 1 fs, corresponding to 10 ps simulation time to reach the equilibrium of the rectangular box. This stabilized rectangular box was used for the main thirty simulations with 5x10^6^ steps (2 fs single step), corresponding to 10 ns for each stabilization using the same simulation parameters as for the box stabilization (see Figure S-3).

Berendsen, H. J. C., J. P. M. Postma, W. F. van Gunsteren, A. DiNola and J. R. Haak (1984). "Molecular dynamics with coupling to an external bath." The Journal of Chemical Physics **81**: 3684-3690.

Darden, T., D. York and L. Pedersen (1993). "Particle mesh Ewald: An N⋅log(N) method for Ewald sums in large systems." The Journal of Chemical Physics **98**: 10089-10092.

Kanai, R., H. Ogawa, B. Vilsen, F. Cornelius and C. Toyoshima (2013). "Crystal structure of a Na+-bound Na+,K+-ATPase preceding the E1P state." Nature **502**: 201-206.

Morth, J. P., B. P. Pedersen, M. S. Toustrup-Jensen, T. L. Sorensen, J. Petersen, J. P. Andersen, B. Vilsen and P. Nissen (2007). "Crystal structure of the sodium-potassium pump." Nature **450**(7172): 1043-1049.

Nyblom, M., H. Poulsen, P. Gourdon, L. Reinhard, M. Andersson, E. Lindahl, N. Fedosova and P. Nissen (2013). "Crystal Structure of Na+, K+-ATPase in the Na+-Bound State." Science **342**: 123-127.
